# Physiomechanical and Surface Characteristics of 3D-Printed Zirconia: An In Vitro Study

**DOI:** 10.3390/ma15196988

**Published:** 2022-10-08

**Authors:** Reem Abualsaud, Maissan Abussaud, Yara Assudmi, Ghadah Aljoaib, Abrar Khaled, Haidar Alalawi, Sultan Akhtar, Asif Matin, Mohammed M. Gad

**Affiliations:** 1 Substitutive Dental Sciences Department, College of Dentistry, Imam Abdulrahman Bin Faisal University, Dammam 31441, Saudi Arabia; 2 Intern, College of Dentistry, Imam Abdulrahman Bin Faisal University, Dammam 31441, Saudi Arabia; 3 Department of Biophysics, Institute for Research and Medical Consultations (IRMC), Imam Abdulrahman Bin Faisal University, Dammam 31441, Saudi Arabia; 4 IRC Membranes & Water Security, King Fahd University of Petroleum & Minerals, Dhahran 31261, Saudi Arabia

**Keywords:** yttria-stabilized tetragonal zirconia polycrystals ceramic, CAD-CAM, 3D printing, flexural strength, mechanical properties, physical properties

## Abstract

The objective of this study is to examine the physiomechanical and surface properties of 3D-printed zirconia in comparison to milled zirconia. A total of 80 disc-shaped (14 × 1.5 ± 0.2 mm) specimens (20 milled and 60 3D-printed (at three different orientations; horizontal, vertical, and tilted)) were manufactured from 3-mol% yttria-stabilized tetragonal zirconia. Five specimens per group were evaluated for crystalline phase, grain size, density, porosity, surface roughness, wettability, microhardness, and SEM analysis of the surface. Biaxial flexural strength (BFS) was measured (*n* = 15) followed by Weibull analysis and SEM of fractured surfaces. Statistical analysis was performed using one-way ANOVA and Tukey’s post hoc test at α = 0.05. All groups showed a predominant tetragonal phase, with a 450 nm average grain size. There was no significant difference between groups with regards to density, porosity, and microhardness (*p* > 0.05). The tilted group had the highest surface roughness (0.688 ± 0.080 µm), significantly different from the milled (*p* = 0.012). The horizontal group presented the highest contact angle (89.11 ± 5.22°), significantly different from the milled and tilted (*p* > 0.05). The BFS of the milled group (1507.27 ± 340.10 MPa) was significantly higher than all other groups (*p* < 0.01), while vertical and tilted had a similar BFS that was significantly lower than horizontal (*p* < 0.005). The highest and lowest Weibull modulus were seen with tilted and milled, respectively. Physical properties of all groups were comparable. The surface roughness of the tilted group was higher than milled. The horizontal group had the highest hydrophobicity. Printing orientations influenced the flexural strength of 3D-printed zirconia. Clinical implications: This study demonstrates how the printing orientation affects the physiomechanical characteristics of printed zirconia.

## 1. Introduction

The application of ceramics in prosthetic and restorative dentistry has grown tremendously in recent years [1]. Dental ceramics play a very crucial role in repairing or replacing lost dental tissues and in the reestablishment of lost functions. Ceramics are highly biocompatible with the oral environment and uphold good mechanical and chemical resistance, in addition to their optical properties that mimic natural teeth [2]. Dental zirconia is extensively used and preferred for prosthetic rehabilitations, especially in areas of high occlusal load due to its qualities in comparison to other ceramic materials, such as superiority in mechanical properties, good aesthetics, sufficient radiopacity, and resistance to microbial adhesion [3]. For dental zirconia, yttrium oxide (Y_2_O_3_, 3 mol%) is added to produce yttria stabilized–tetragonal zirconia polycrystal (3Y-TZP) [1]. Y-TZP is unique in the ability to undergo transformation toughening by changing phases from tetragonal phase to monoclinic phase upon stress induction. This transformation is associated with an increase in crystal structure volume that helps in ceasing crack propagation [4,5].

The increased demands for these new ceramic restorations over the past years resulted in a great advancement of the existing systems or the introduction of new restorative techniques [6]. Ceramic crowns are primarily manufactured digitally using the subtractive method (SM), i.e., milling to remove excess material from ceramic blocks with the help of computer-aided designing and computer-aided manufacturing (CAD/CAM) [7]. However, this technique presents several manufacturing limitations including restricted capability to create structures with complex internal geometry, and waste material during production [7,8]. Additionally, the milling tools show signs of wear with progressive use. Therefore, the number of milling procedures per milling tool should not exceed what is prescribed by the manufacturer guidelines to prevent bur fracture [9]. The material being milled also influences the durability of the burs. The harder the substrate, the more loss of diamond is anticipated. This in return affects the surface topography of the milled restoration [10]. To add to that, the clinicians must educate themselves on the milling bur limitations (diameter/thickness, taper, and length) during the manufacturing of a prosthesis in order to include certain modifications within tooth preparation to avoid over-milling or under-milling [11]. 

To overcome the drawback of SM, additive manufacturing (AM) has been rapidly developing. AM offers multiple advantages in comparison to SM techniques. It involves building an object with consecutive thin layers that are either cured as they are built or receive post-layering curing. This approach of manufacturing uses CAD software and a 3D printer which are essential elements in the viewing of planned objects in a virtual environment and then producing the actual object [2,12].

The 3D-printing technology has brought many advancements into the dental field by improving the processing, and increasing the dental applications of polymers, ceramics, and metals within the dental treatment options [13,14,15]. Since the introduction of zirconia into the dental field, it has been conventionally manufactured through SM. However, in recent years, there has been an increasing trend in producing different zirconia objects thought AM techniques (3D printing) [16]. Early attempts showed some degree of success in producing dental prostheses and implants. However, they were not free from surface pores, microcracks, and uneven shrinkage [16]. Current AM methods to fabricate ceramic objects, in general, include stereolithography (SLA), selective laser sintering (SLS), multi-jetting, and material extrusion, among others [7,15]. The SLA method has been used successfully in the biomedical field to build polymeric surgical templates and maxillofacial prostheses. In this technique, the object is built in the green stage by layering through selective curing of a photosensitive binder, followed by sintering to achieve the final density [17,18]. However, AM of ceramics still carries few limitations related to raw material homogeneity, printing parameters, and post-printing processing [19].

Furthermore, the mechanical and surface properties of materials produced by 3D printing is said to be affected by the printing technique [20,21] and printing orientation [22,23]. Printing orientation is one of the primary variables that affect AM output and is also a prime parameter at the initial stage due to its effects on object precision/trueness and quality [22]. Additionally, the build direction of layers affects the surface geometry and thereafter influences the surface properties [22,23]. By the same token, mechanical, esthetic, and biological properties have also been affected by printing orientation. This has been reported extensively for polymers [22,23,24,25,26,27,28]. Similar studies are not yet available at such wide scale for dental ceramics. The investigated variables of AM ceramics included material composition [29,30], porosity [8], or the comparison of AM zirconia to that of the SM zirconia [3,29,31,32,33]. Only a scarce number of studies compared between the properties of horizontally and vertically printed ceramics [29,34,35]. 

Examining the microstructure and basic physical properties of these 3D-printed zirconia materials will enable us to determine the dependability and trueness of fabricating zirconia crowns using the AM process. Despite the reported potential success of the AM technique for ceramics, it is still in its initial stage of development. The purpose of this study was to evaluate the mechanical, physical, and surface properties of 3D-printed yttria–partially stabilized zirconia material and compare the results with those of milled zirconia specimens. The null hypotheses of the study are as follows: First, the different printing orientations will not affect the tested properties; crystalline structure, grain size, density, porosity, surface roughness, wettability, microhardness, and biaxial flexural strength (BFS) of 3D-printed zirconia. Second, 3D-printed zirconia specimens would have comparable results to those of milled specimens.

## 2. Materials and Methods

Based on the effect size detected between AM and SM zirconia [29,32] and that between horizontally and vertically printed zirconia specimens [36], with a level of significance set at 0.05 and a power of 95%, each test group must contain 12 specimens (https://clincalc.com/stats/samplesize.aspx, accessed on 7 September 2021). However, the number of specimens dedicated for BFS was increased to 15 to allow for Weibull analysis later as recommended by ISO standard #6872 [37]. Additionally, 5 specimens per group were used to test other properties including density, porosity, surface roughness, wettability, and microhardness. Out of these, 2 representative specimens per group were used for SEM analysis, crystalline phase distribution, and grain size measurement. This resulted in 80 total specimens, 60 for BFS (15/milled and 45/3D-printed, 15/orientation) and 20 for other properties (*n* = 5). All specimens were manufactured from 3 mol% yttria–partially stabilized tetragonal zirconia polycrystals (3Y-TZP), Table 1, made either through SM or AM technologies. Disc-shaped specimens with the desired dimensions (diameter 14 mm, thickness 1.5 ± 0.2 mm) as recommended by ISO standard #6872-2015 [37] were designed digitally using CAD software (123D design, Autodesk, version 2.2.14, Mill Valley, CA, USA) then exported as an STL file. SM specimens were milled out of zirconia disc (IPS e.max ZirCAD LT, thickness 18 mm and shade A2-Ivoclar Vivadent AG, Schaan, Liechtenstein) using a 5-axis CAM machine (PrograMill PM7, Ivoclar Vivadent AG). 

The specimens were milled in the green stage and therefore were sintered according to manufacturer’s directions. 

**Figure 1:**
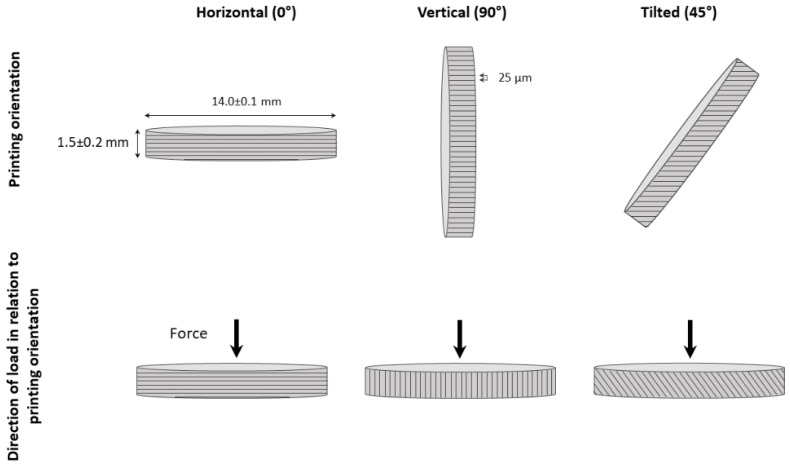
Schematic representation of the specimen printing orientation and relation to the applied load.

For AM specimens, the same STL file was sent to a commercial supplier to 3D print and sinter the specimens. The 3D-printed discs were produced out of 3Y-TZP paste (3DMix, 3DCeram Sinto) using a 3D printer (CERAMAKER C900 Flex, 3DCeram Sinto), Table 1, at three different printing orientations: horizontal (0°), titled (45°), and vertical (90°) in relation to printing platform, Figure 1, with a printing layer thickness of 25 µm, and 1.25–1.28× scaling of final specimen dimensions. The four test groups are referred to as milled, horizontal, tilted, and vertical.

The specimens were tested as received from the manufacturer with no further modification except polishing of the specimens undergoing BFS. These specimens were polished using 1200-grit silicon carbide paper (MicroCut PSA; Buehler, IL, USA) followed by 3 μm polishing suspension (MetaDi Supreme, Polycrystalline diamond suspension 3 μm, Buehler GmbH, IL, USA) combined with a polishing cloth and machine (Metaserv 250 grinder-polisher; Buehler GmbH, IL, USA). Polishing of all specimens was completed by a single operator and the dimensions of the specimens were confirmed after polishing using an electronic digital caliper (NEIKO 01407A Electronic Digital Caliper, Neiko tools USA, Greenacres, FL, USA). The specimens used to test the physical and surface properties were cleaned in an ultrasonic bath and air dried for 24 h between tests, and each test was completed by a single examiner.

### 2.1. Microstructure, Crystalline Phase Distribution, and Grain Size (nm)

These properties were measured using X-ray diffraction (XRD) (Shimadzu XRD-7000, with Cu Kα radiation (λ = 1.5406 Å)). The samples (*n* = 2) were scanned between 2θ = 20–90° at a scan rate of 0.5°/min. The interplanar spacing was calculated using Bragg’s law while the crystallite size was found using Scherrer’s formula.

### 2.2. Density (g/cm^3^) and Apparent Porosity (%)

Density was determined using the Archimedes method [30,38], where the specimens (*n* = 5) were dried in an oven at 100 °C for 1 h and then weighed individually using an analytical digital lab balance (LEADZM B3003T, Leadzm, New Orleans, LA, USA) with 0.001 g accuracy. Following that, the weight was recorded for the specimen after suspending in distilled water at 23 °C. Density was calculated using the following Formula (1):

(1) $$D=\frac{m_{1}}{m_{1}-m_{2}}\times D_{w}$$

*D* is the density of the specimen (g/cm^3^); *m*_1_ is the mass of dry specimen (g); *m*_2_ is the mass of the suspended specimen; and *D_w_* is the density of the immersion liquid. To calculate the apparent porosities (*n* = 5), the following Formula (2) was used [38]:

(2) $$P=\frac{m_{3}-m_{1}}{m_{3}-m_{2}}\times 100$$

where *P* is the apparent porosity (%) and *m*_3_ is the mass of the soaked specimen (g).

### 2.3. Surface Roughness, R_a_ (µm)

Measurements of the surface roughness (R_a_) (*n* = 5) were undertaken at 5 separate locations, 1 at the center and 4 circumferentially on each specimen using a non-contact optical profilometer (Contour GT; Bruker Nano GmbH, Berlin, Germany). The R_a_ value was averaged per specimen then per group [39].

### 2.4. Wettability/Contact Angle (°)

The wettability was determined using the sessile drop method [39] (*n* = 5). Measurements of the contact angles were performed at 20 °C using a goniometer (DM-501; Kyowa Interface Science Co., Niiza, Japan) with a video camera. A drop of distilled water (10 µL) was generated on the sample’s surface using a pipette. Following that, images of the water drop were captured 15 s after the application and interpreted using software (FAMAS, Kyowa Interface Science Co., Japan). The process was repeated three times for each specimen, then the values were averaged to calculate the contact angle per specimen and group [39].

### 2.5. Microhardness (VHN)

Vickers indenter (MicroMet 6040, Buehler, IL, USA) was used to measure the microhardness (*n* = 5). Five indentations were made circumferentially in each disc using 9.81 N for 15 s. The average hardness value per specimen was calculated followed by averaging to obtain the group hardness value [3].

### 2.6. Biaxial Flexural Strength (MPa) and Weibull Modulus

The specimens (*n* = 15) were individually placed on a supporting jig made of three hardened steel balls equally spaced on the circumference of a circle (120°). A universal testing machine (Instron 5965, Instron, MA, USA) equipped with 5 KN load cell applied the load at the center of the specimen using a 1.5 mm flat-end piston, at a crosshead speed of 0.5 mm/min until specimen fracture. A piece of rubber spacer (thickness 0.2 mm) was placed between the specimen and the load. The load at fracture was recorded per specimen and the BFS was calculated using the following Formula (3) [37]:

(3) $$\sigma =-0.2387P\left(X-Y\right)∕b^{2}$$

where *σ* is maximum flexural strength (MPa), *P* is maximum load causing fracture in (N),

(4) $$X=\left(1+v\right)In{\left(\frac{r_{2}}{r_{3}}\right)}^{2}+\left[\frac{1-v}{2}\right]{\left(\frac{r_{2}}{r_{3}}\right)}^{2},$$

(5) $$Y=\left(1+v\right)\left[1+In{\left(\frac{r_{1}}{r_{3}}\right)}^{2}\right]+\left(1-v\right){\left(\frac{r_{1}}{r_{3}}\right)}^{2},$$

v is Poisson’s ratio (0.3), $r_{1}$ is the support circle radius, $r_{2}$ is the loaded area radius, $r_{3}$ is the specimen radius, and *b* is the thickness of specimen at fracture, all in mm. 

### 2.7. SEM Analysis

The surface morphology of the intact specimens (*n* = 2) and fractured specimens (*n* = 2) was studied using scanning electron microscopy (SEM) (FEI, Inspect S50, Brno, Czech Republic at 20 kV). The samples were mounted onto an SEM stub using double-sided carbon tape. The SEM was operated at 20 kV and images of the gold-coated specimens were taken at multiple magnifications to highlight the various features and to disclose the grains of the specimens. However, representative images were displayed at magnifications of 500, 5000, and 20,000× for the as-received specimens, while fractured specimens were shown at 200 and 1000×.

JMP 16 (SAS, Cary, NC, USA) was used to perform all statistical analyses. Means and standard deviations were calculated. Following that, appropriate tests were used. A one-way ANOVA per tested property was run and when significant, Tukey’s post hoc test was employed to study pairwise comparisons. The level of significance was set at 0.05 for all tests.

## 3. Results

The mean values ± SD, of the tested properties (density, porosity, surface roughness, wettability/contact angle, microhardness, BFS, Weibull modulus) are summarized in Table 2. 

### 3.1. Crystalline Phase Distribution and Grain Size

**Figure 2:**
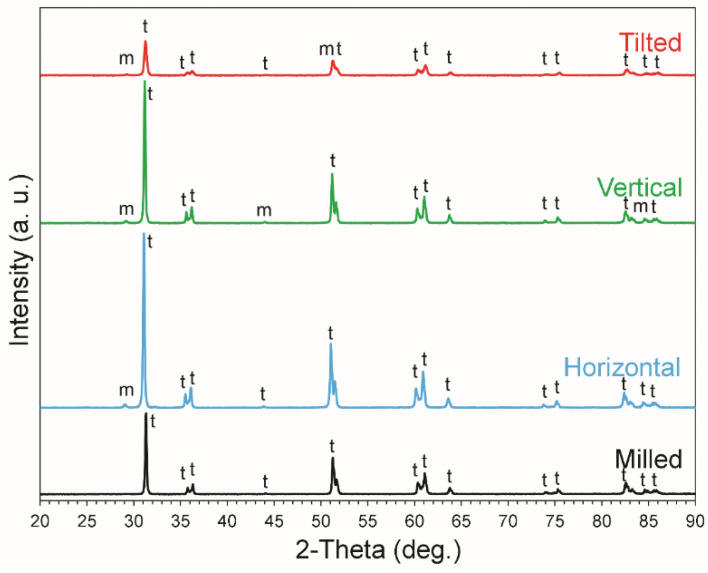
The XRD pattern of representative specimens from each group (m: monoclinic phase; t: tetragonal phase).

According to the XRD analysis, Figure 2 shows the pattern for representative zirconia specimens from each group. The tetragonal phase was the principal phase detected in all groups, with varying percentages of monoclinic phase. The milled specimens lacked the monoclinic phase compared to 3D-printed groups. The horizontal, vertical, and tilted specimens presented with 0.93, 2.95, and 14.2% of monoclinic phase, respectively. The average grain size ranged between 418 and 458 nm in all groups. With regards to crystalline size at maximum intensity of XRD, the milled, horizontal, and vertical groups had nearly double the crystalline size of the titled group (Table 2).

### 3.2. Density (g/cm^3^) and Apparent Porosity (%)

The densities of the specimens in the four groups were not significantly different from each other (ANOVA results; *F* = 0.333, *p* = 0.802). The highest and lowest densities were reported with milled (6.056 ± 0.116 g/cm^3^) and tilted (5.942 ± 0.266 g/cm^3^), respectively. The apparent porosity of the specimens ranged between 0.923 ± 0.591% and 1.945 ± 1.509% for milled and tilted with no significant difference between the groups (ANOVA results; *F* = 1.27, *p* = 0.318).

### 3.3. Surface Roughness, R_a_ (µm)

**Figure 3:**
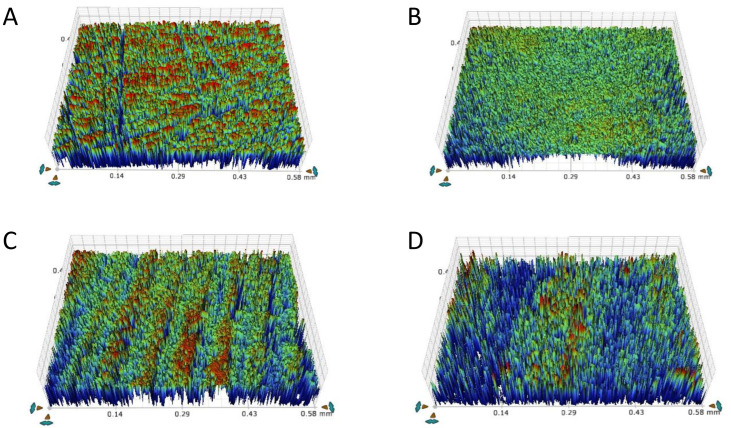
Images of surface roughness of specimens. (**A**) Milled, (**B**) horizontal, (**C**) vertical, and (**D**) tilted.

The highest and lowest surface roughness values were reported with tilted and milled, respectively. The surface roughness of the specimens varied significantly (ANOVA results; *F* = 4.356, *p* = 0.02). The tilted group had the highest R_a_ (0.688 ± 0.080 µm), significantly different (*p* = 0.017) from milled (0.542 ± 0.087 µm). However, no difference was detected between the milled and the other two printed groups (*p* > 0.05), Figure 3.

### 3.4. Wettability/Contact Angle (°)

**Figure 4:**
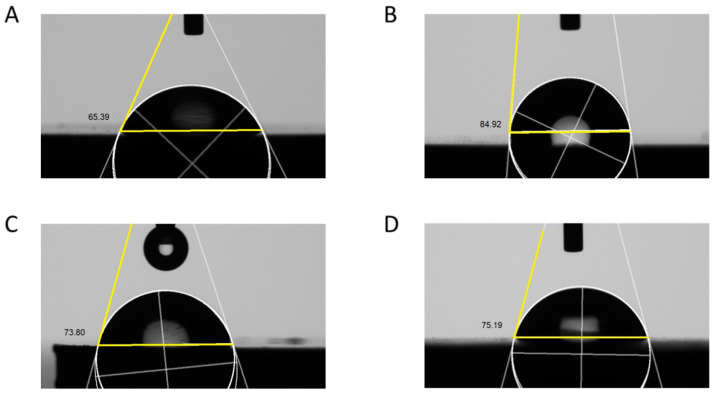
Images of contact angles of the specimens. (**A**) Milled, (**B**) horizontal, (**C**) vertical, and (**D**) tilted.

The ANOVA results showed that the contact angles (wettability) of the specimens differed significantly between the groups (ANOVA results; *F* = 4.957, *p* = 0.013). Pair-wise comparisons using Tukey’s post hoc test revealed a significantly higher contact angle with horizontal (89.11 ± 5.22°) than milled (*p* = 0.011) and tilted (*p* = 0.047), but not vertical (75.34 ± 9.24°). The vertical group was not different from milled or tilted either (*p* > 0.05), (Figure 4).

### 3.5. Microhardness (VHN)

With regards to surface hardness, all groups showed similar hardness values with no significant difference (ANOVA results; *F* = 2.556, *p* = 0.092). The highest hardness value was reported for horizontal (1676.61 ± 37.77 VHN1) and the lowest for milled (1548.2 ± 62.32 VHN1). 

### 3.6. Biaxial Flexural Strength (MPa) and Weibull Modulus

For BFS, the milled and vertical groups showed the highest and lowest values at 1507.27 ± 340.10 MPa and 521.51 ± 88.76 MPa, respectively. The ANOVA results indicated a significant difference among the groups (ANOVA results; *F*= 36.3176, *p* < 0.001), and the post hoc test revealed significant differences between milled vs. all other groups (*p* < 0.01), horizontal vs. all other groups (*p* < 0.01), while vertical and tilted were not significantly different from each other (*p* = 0.11). 

**Figure 5:**
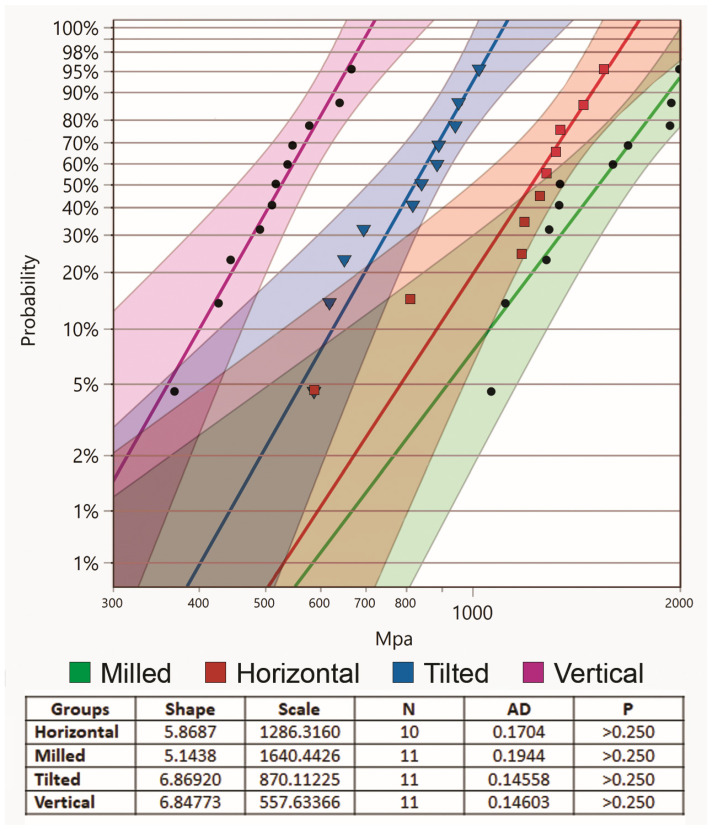
Weibull modulus.

The Weibull modulus presented the highest shape for tilted (6.8692) and the lowest for milled (5.1438), with vertical and horizontal ranking second and third, respectively, Figure 5.

### 3.7. SEM Analysis

#### 3.7.1. Initial Analysis

**Figure 6:**
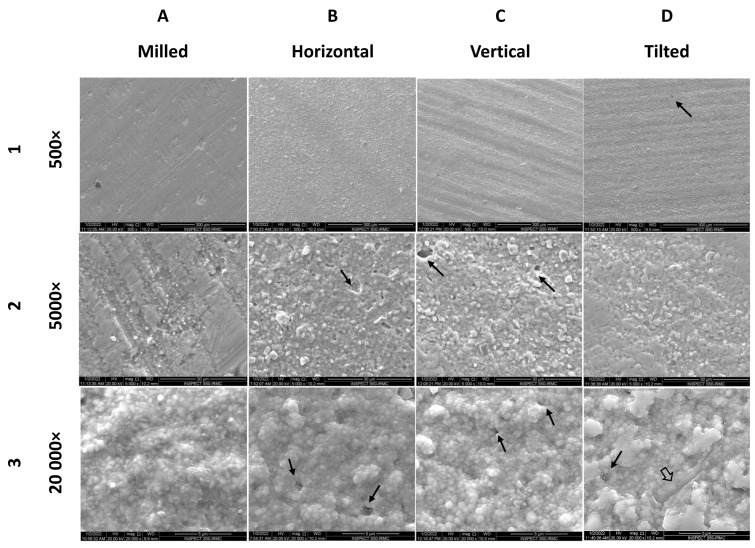
Surface characteristics of representative specimens from each group (**1A**–**1D**) at 500×, (**2A**–**2D**) at 5000×, and (**3A**–**3D**) at 20,000×.

The SEM images of the as-manufactured specimens from each group are displayed in Figure 6 at different magnifications. At low magnification (500×), the milled and horizontal specimens show dense and smooth surfaces with some irregularities (representing scratches of the milling bur (Figure 6(1A)) or clusters of zirconia grains (Figure 6(1B)). While for vertical (Figure 6(1C)) and tilted (Figure 6(1D)), the surface clearly shows bands of parallel waves of zirconia representing the layers of printing. 

The higher magnification (5000×) of printed specimens’ surfaces showed clustering of zirconia grains into clumps in addition to the presence of few voids on the surface that ranged between 2 and 10 microns across (see black arrows). 

After further magnification (20,000×), the milled specimens showed a dense structure with no voids and slightly polygonal grains in the range of 270–760 nm and an average of 450 nm. Smaller voids are detected in printed specimens (Figure 6(3B–3D) with rounded grains in the range of 240–920 nm. In addition, the tilted specimen shows deep and wide grooves, as marked by a hollow black arrow with a slight fusion of smaller grains into larger clumps (Figure 6(3D)).

Voids are marked with black solid arrows. Crack lines are marked with a hollowed arrow.

#### 3.7.2. SEM Analysis of Fractured Specimens

**Figure 7:**
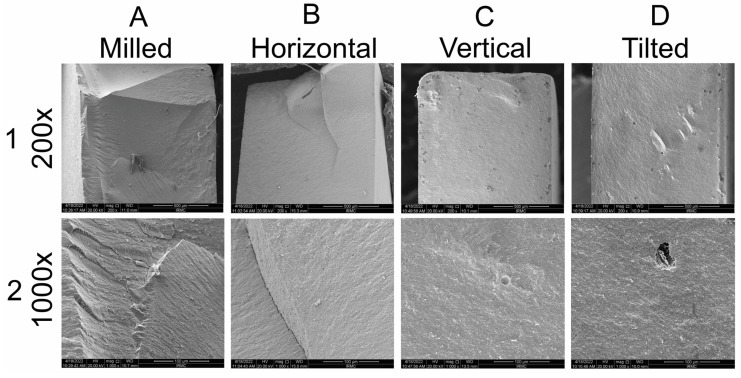
Surface of fractured specimens at (**1A**–**1D**) 200× and (**2A**–**2D**) 1000×.

Images of fractured specimens at 200× and 1000× are shown in Figure 7. Crack lines were seen extending from the surface inward with smaller crack lines scattered throughout the specimen (Figure 7(1A,2A)). The horizontal group showed a dense structure with cracks extending parallel to the printing orientation (Figure 7(1B,2B)). Vertical and tilted groups showed a homogenous structure with minor voids as seen in Figure 7(1D,2C,2D). 

## 4. Discussion

Comparisons of the available techniques (SM and AM) to fabricate zirconia restorations, the different printing orientations, and the determination of possible clinical uses and limitations are difficult, due to the limited publications on this topic. This study inspected and analyzed surface characteristics along with physical and mechanical properties of zirconia ceramic (3Y-TZP) manufactured by two different techniques: subtractive and additive. The results of the study suggested partial rejection of the first hypothesis with regards to contact angle and BFS. The second hypothesis was also partially rejected for roughness, contact angle, and BFS. 

Since the future is advancing rapidly in the field of 3D printing of restorations, soon, this technology will be available widely for ceramic restoration on a commercial level. Therefore, this study aimed to characterize one of the currently available zirconia materials and test a number of its properties that are relevant to the oral environment. 

According to the XRD analysis, the present study demonstrated that all zirconia groups (SM and AM) had comparable phase composition and pattern with tetragonal phase forming the bulk of the material, similar to what was reported by Nakai et al. [29]. However, in our study, monoclinic phase was detected more apparently in the tilted group. Similarly, the absence of monoclinic phase in the milled group was also reported by Moqbel et al. [4]. The zirconia-phase composition is determined by the yttria content of the zirconia powder in the printing slurry [29], and the surface treatment employed after sintering such as airborn particle abrasion which might initiate phase transformation [4]. However, in our study, specimens did not receive any surface treatment.

All groups presented a grain size in the range of 240–920 nm with average of 420–460 nm. Although all printed groups received similar manufacturing steps, debinding and sintering temperatures, a slight difference in grain size was noted. These findings were aligned with Hofer et al. [30], who indicated an average grain size of 0.43–0.45 µm. The slight discrepancy regarding the range of size between the studies might be related to the difference in materials used (LithaCon3Y230 and LithaCon3Y210 with 44% and 48% solid loading, respectively), original grain size (0.4 µm) [30], or the debinding and sintering temperatures [40,41].

The surface porosities/voids seen in all printed groups might be linked to the manufacturing technique involving layering the specimen in thin sections which could entrap air during the process. This finding is inconsistent with Hofer et al. [30], who reported the absence of voids upon SEM analysis of printed specimens. However, Li et al. [42] suggested the debinding process in the stereolithography manufacturing of zirconia specimens to be one of the causes for void formation. Whereas the milled specimens showed a dense structure with no voids as a result of the isostatic pressing of the zirconia disc and multidirectional scratches of different widths and depths indicative of the effect of milling burs during machining. These findings were contrary to what was reported by Baysal et al. [3] confirming more pores on the surface of the milled specimens compared to printed. 

Density is an important factor that influences the mechanical and optical properties of the material [8,42]. The test groups in this study showed similar density values. The milled and tilted groups had the highest and lowest densities, respectively. Those values were similar to previous studies [2,34,35]. A published article [40] concluded that grain size influences the material density. The larger the grains, the denser the material. Thus, supporting the finding of the current study where the tilted group showed a slightly smaller average grain size and lower density. Considering the density of tetragonal zirconia to be 6.10 g/cm^3^ [43,44], the relative densities of all groups were above 97%. Whereas the apparent porosities were consistent with those reported by a previous study for milled, horizontal, and vertical groups [34]. The apparent porosity of the titled group showed the highest value in comparison to other groups. However, the values were not significantly different from each other. Harrer et al. [35] suggested that lowering the viscosity of the raw material, modifying the binder or printing parameters, and degassing of the ceramic paste, could reduce the resulting porosities. 

The aggregation of the nano-sized grains to form clumps has been seen in SEM images of AM specimens. This has been reported earlier by Li et al. [19], who related that to the nature of the nanoparticles and the high surface energy that promotes aggregation. Therefore, it was suggested to limit the printed layer thickness to 25 µm for better surface finish and precision which was followed in this study. Surface roughness is an important influencer of the esthetic results of a restoration [42], as well as the amount of opposing tooth/restoration wear [1]. All the printed specimens showed similar surface roughness with the horizontal being the smoothest of the three. These findings were slightly different than those reported by Schiltz et al. [34] in value and trend for the as-received surface roughness. One might explain the slight increase in surface roughness for vertical and tilted by the direction of roughness measurement in relation to layer orientation and the presence of steps between consecutive layers in these two groups compared to horizontal. The surface of the milled group was smoother than the printed groups with minor milling bur scratches, conforming with the roughness values for that group. The roughness values obtained in this study for all groups were much higher than the maximum acceptable roughness value (0.2 µm), which might increase the risk of microbial adhesion and plaque accumulation [45]. However, those values were lower than Osman et al. for printed specimens [36], but higher than Branco et al. [2] and Baysal et al. [3] for milled or printed specimens. The discrepancy between this study and Branco’s could be related to the difference in measuring technique (contact vs. optical vs. AFM) or the use of different materials for SM or AM. In their study, the grain size of the SM and AM zirconia was more towards the higher range (~500–700 nm) compared with our study. In addition, the layer thickness was much higher (0.2 mm), and their specimens underwent polishing with 3 µm diamond paste before roughness measurement, while in this study, the surface roughness was recorded for the specimens as received. 

The wettability of the surface has been linked to the surface topography and charge. Additionally, greater cell adhesion is linked to higher surface wettability and surface energy [39]. The results of this study pointed to more favorable wetting with milled specimens compared to horizontal, while titled and vertical groups were comparable to both groups. The wetting of the milled group was close to that reported by Noro et al. [39]. In contrast, the findings of this study were opposing to Branco et al. [2] who reported a lower contact angle with printed specimens. In their study, they subjected the specimens to polishing before testing. 

The hardness of a material represents its ability to resist plastic deformation upon indentation force [46]. In our study, all groups showed similar microhardness values regardless of the production technique or orientation. These values were higher than previous studies [4,30,31]. The difference may be due to the use of a different measuring unit, modification in binder and zirconia loading [30], the use of translucent zirconia (4.5%–≤6.0% yttria) [4], or the difference in production technique [31]. Baysal et al. [3] reported similar hardness values for the milled group (1501.4 ± 60.1 VHN), but lower values for the printed group (1169.2 ± 48.4 VHN) in comparison to ours. Branco et al. [2] and Harrer et al. [35] also reported less hardness for AM groups irrespective of measuring direction or printing orientation. 

Since the current investigations of the 3D-printed zirconia will lay the foundation for future advancements and improvements to the material or technique before full adoption into the production streamline, it is very important to test the flexural properties of the 3D-printed material in different printing orientations and compare the results to those of SM. Accordingly, milled and vertical groups had the highest and lowest BFS, respectively. Those findings are consistent with previous reports [3,47]. The high strength of the milled group might be explained by the manufacturing techniques and the structure of the resulting specimens after sintering with densely packed grains, higher relative density, and absence or minimal internal porosities or defects. In contrast, among the printed specimens, the horizontal group showed the highest BFS. One possible reason is the number of layers in each printed specimen. The horizontal specimen has a lower number of layers forming the final shape compared to tilted or vertical. Although distinct demarcation between consecutive layers was not observed within the bulk of specimen in SEM images, this variable may influence the number and volume of defects and voids between layers and hence the final specimen strength. Previous reports indicated that the build orientation of the specimen in relation to the applied load imposes an effect on the strength of the specimen, where specimens built in layers perpendicular to the applied load showed higher strength values [29,34]. In contrast, a few other studies reported higher flexural strength values for AM zirconia specimens compared to SM specimens [8,32], or comparable flexural strength values of the two manufacturing techniques [29,48]. Those differences might be related to different tests used (uniaxial vs. biaxial testing), material composition, or printing technology (DLP vs. SLA). Moreover, the tilted and vertical groups showed a lower percentage of tetragonal zirconia, which lowers its ability to undergo transformation into monoclinic to stop the crack propagation, resulting in lower strength. Regardless of the vertical group showing the lowest BFS, the results of all tested groups were above the minimum recommended value (500 MPa) [37] for monolithic full-contour, partially, or fully covered substructures for single or three-unit prostheses involving the molars. When four or more units are needed, horizontal, tilted, or milled prostheses are recommended. 

Weibull analysis was used to analyze the consistency of the tested specimen. The higher the value, the less variation is present between specimens [8]. The highest and lowest calculated Weibull moduli were seen with tilted and milled groups, respectively. Those findings are similar to one study [29], but the reverse of multiple others [8,32,36,47]. However, according to the ISO standard #6872-2015 [37], at least 15 specimens per group are needed for reliable Weibull analysis. This study aimed at testing 15 specimens per group, however, outliers were encountered in all groups and had to be eliminated during statistical analysis. Therefore, the results of the Weibull analysis are deficient and should not be used to draw a conclusion.

At a clinical level, the produced restoration/crown must fulfil the mechanical and biological requirements as well as have good esthetics. According to the results of this study, the clinician must consider the most important feature/property of the restoration to match it with the appropriate printing orientation. If strength was the prime concern, a horizontally printed restoration is recommended, while it should be avoided if high wettability was the top interest of the dentist. However, there are other aspects of a material that are also considered prime for its success but could not be tested in this study. For example, the restoration must be accurate and precisely duplicate the original design in order to minimize the need for chair-side corrections. Alharbi et al. [22] and Osman et al. [23] studied the effect of printing orientation on the accuracy of SLA- and DLP-printed resin crowns. They concluded that 120° and 135° printing orientations (180° is when supports are located at occlusal surface), respectively, provided the most accurate crown. It is still not known whether the same results will be obtained if the material of manufacture was zirconia. Few studies [18,49,50] reported the accuracy and trueness levels of printed zirconia crowns in comparison to milled ones. However, none has specified the printing orientation of their specimens. In this study, measuring the accuracy of the printed discs was not one of the objectives. Therefore, the authors recommend further investigations to evaluate the effect of printing orientation on the accuracy and trueness of 3D-printed zirconia before making a final judgement on the best angle to use in clinical settings. 

The study aimed to characterize AM zirconia specimens printed at three different orientations and compare the results to those of SM zirconia. The findings play an important role in determining the most appropriate printing orientation for favorable mechanical and surface properties of the final prosthesis. However, among the encountered limitations is the use of only one type of zirconia per manufacturing technique, the limited number of tests, the use of standardized specimens that do not represent actual restorations, the absence of aging, in addition to the limited published papers involving different orientations of AM zirconia, which made comparisons with previous reports more difficult. Based on that, future studies are encouraged to test more zirconia brands, include more printing orientations, test specimens with a geometry closer to a dental restoration, test aspects related to color and translucency, evaluate microbial adhesion, test the strength of bonding to ceramic, and involve the specimens in aging and wear processes, in addition to the evaluation of the trueness/accuracy of different printing orientations. 

## 5. Conclusions

The 3D-printing orientation had an influence on the wettability and biaxial flexural strength of 3D-printed zirconia. However, all 3D-printed specimens achieved the minimum required strength for single (monolithic or substructure) restoration involving molars as recommended by ISO. Physical properties (density, porosity, and micro-structure), and microhardness of all groups were comparable to each other. The roughness value of tilted specimens was higher than milled, while the wettability of horizontally printed specimens was the lowest. 

## Figures and Tables

**Table 1:** Details of zirconia used in this study.

Group	Material	Manufacturer	Technology	Sintering Protocol	Composition *		Orientation
Subtractive Manufacturing (SM)	IPS e.max ZirCAD LT	Ivoclar vivadent AG	Dry milling using a 5-axis milling machine (PM7)-	Immediate sintering at max. temp 1500 °C for 9 h	Element/compound	wt%	Vertical within the disc
					ZrO_2_	88.0–95.5	
					Y_2_O_3_	>4.5–≤6.0	
					HfO_2_	≤5.0	
					Al_2_O_3_	≤1.0	
					Other oxides for coloring	≤1.0	
Additive Manufacturing (AM)	3DMix ZrO_2_	3DCeram	Stereo-lithography using a 3D-printer (CERAMAKER C900 Flex, 3DCeram Sinto, France)	Debinding by raising temp. slowly to 1000 °C then cooling slowly. Followed by sintering at max. temp. 1450 °C for ~ 20 h	Element	ppm	Horizontal (0°) Tilted (45°) Vertical (90°)
					Li	0.21	
					Na	9.2	
					Mg	9.2	
					Al	~2800	
					Si	380	
					Y	Matrix	
					Zr	Matrix	
					Ag	<200	
					Cd	<200	
					Hf	~21,000	
					Ta	<10	

* The chemical analysis was provided by the manufacturers.

**Table 2:** Mean (SD) and significance between groups per tested properties.

Property	Milled Group	3D-Printed Groups			*p*-Value
		Horizontal	Vertical	Tilted	
Density (g/cm^3^)	6.065 (0.116)	5.978 (0.061)	5.987 (0.223)	5.942 (0.266)	0.802
Apparent porosity (%)	0.923 (0.591)	0.948 (1.086)	0.970 (0.350)	1.945 (1.509)	0.318
Roughness (µm)	0.542 (0.087) ^B^	0.626 (0.043) ^AB^	0.660 (0.046) ^AB^	0.688 (0.080) ^A^	0.020 *
Wettability/Contact angle (°)	69.41 (13.18) ^A^	89.11 (5.22) ^B^	75.34 (9.24) ^AB^	73.39 (3.1) ^A^	0.013 *
Hardness (VHN)	1548.2 (62.32)	1676.61 (37.77)	1609.54 (87.55)	1634.96 (98.1)	0.092
Biaxial flexural strength (MPa)	1507.27 (340.10) ^A^	1186.73 (283.47) ^B^	521.51 (88.76) ^C^	810.92 (148.84) ^C^	<0.001 *
Average grain size- measured from SEM (nm) !	448 ± 97.1	420 ± 151.8	458 ± 152.0	418 ± 139.1	-
Crystalline size (I_max_)- measured from XRD (nm) !	96	96	96	57	-

Similar capital superscripted letters indicate no significant difference between groups property-wise. * Indicates a significant difference at α = 0.05. ! Descriptive statistics only.

## Data Availability

The data presented in this study are available on request from the corresponding author.

## References

[1] Botelho M.G., Dangay S., Shih K., Lam W.Y.H. (2018). The effect of surface treatments on dental zirconia: An analysis of biaxial flexural strength, surface roughness and phase transformation. J. Dent..

[2] Branco A.C., Silva R., Santos T., Jorge H., Rodrigues A.R., Fernandes R., Bandarra S., Barahona I., Matos A.P.A., Lorenz K. (2020). Suitability of 3D printed pieces of nanocrystalline zirconia for dental applications. Dent. Mater..

[3] Baysal N., Tuğba Kalyoncuoğlu Ü., Ayyıldız S. (2022). Mechanical Properties and Bond Strength of Additively Manufactured and Milled Dental Zirconia: A Pilot Study. J. Prosthodont..

[4] Moqbel N.M., Al-Akhali M., Wille S., Kern M. (2020). Influence of Aging on Biaxial Flexural Strength and Hardness of Translucent 3Y-TZP. Materials.

[5] Guilardi L.F., Pereira G.K., Wandscher V.F., Rippe M.P., Valandro L.F. (2017). Mechanical behavior of yttria-stabilized tetragonal zirconia polycrystal: Effects of different aging regimens. Braz. Oral Res..

[6] Kashkari A., Yilmaz B., Brantley W.A., Schricker S.R., Johnston W.M. (2019). Fracture analysis of monolithic CAD-CAM crowns. J. Esthet. Restor. Dent..

[7] Revilla-León M., Methani M.M., Morton D., Zandinejad A. (2020). Internal and marginal discrepancies associated with stereolithography (SLA) additively manufactured zirconia crowns. J. Prosthet. Dent..

[8] Zandinejad A., Das O., Barmak A.B., Kuttolamadom M., Revilla-León M. (2022). The Flexural Strength and Flexural Modulus of Stereolithography Additively Manufactured Zirconia with Different Porosities. J. Prosthodont..

[9] Roperto R.C., Lopes F.C., Porto T.S., Teich S., Rizzante F.A.P., Gutmacher Z., Sousa-Neto M.D. (2018). CAD/CAM diamond tool wear. Quintessence Int..

[10] Al Hamad K.Q., Al Quran F.A., Jwaied S.Z., Al-Dwairi Z.N., Al-Rashdan B.A., Baba N.Z. (2022). Effect of CAD/CAM Bur Deterioration on the Surface Roughness of Ceramic Crowns. J. Prosthodont..

[11] Turkyilmaz I., Wilkins G.N., Yun S. (2022). Moving from analogue to digital workflows in dentistry: Understanding undermilling and overmilling as detrimental factors in fabricating CAD/CAM crowns. Prim. Dent. J..

[12] Dawood A., Marti Marti B., Sauret-Jackson V., Darwood A. (2015). 3D printing in dentistry. Br. Dent. J..

[13] Schweiger J., Edelhoff D., Güth J.F. (2021). 3D Printing in Digital Prosthetic Dentistry: An Overview of Recent Developments in Additive Manufacturing. J. Clin. Med..

[14] Barazanchi A., Li K.C., Al-Amleh B., Lyons K., Waddell J.N. (2017). Additive Technology: Update on Current Materials and Applications in Dentistry. J. Prosthodont..

[15] Pillai S., Upadhyay A., Khayambashi P., Farooq I., Sabri H., Tarar M., Lee K.T., Harb I., Zhou S., Wang Y. (2021). Dental 3D-Printing: Transferring Art from the Laboratories to the Clinics. Polymers.

[16] Khanlar L.N., Salazar Rios A., Tahmaseb A., Zandinejad A. (2021). Additive Manufacturing of Zirconia Ceramic and Its Application in Clinical Dentistry: A Review. Dent. J..

[17] Li R., Wang Y., Hu M., Wang Y., Xv Y., Liu Y., Sun Y. (2019). Strength and Adaptation of Stereolithography-Fabricated Zirconia Dental Crowns: An In Vitro Study. Int. J. Prosthodont..

[18] Wang W., Yu H., Liu Y., Jiang X., Gao B. (2019). Trueness analysis of zirconia crowns fabricated with 3-dimensional printing. J. Prosthet. Dent..

[19] Li H., Song L., Sun J., Ma J., Shen Z. (2019). Dental ceramic prostheses by stereolithography-based additive manufacturing: Potentials and challenges. Adv. Appl. Ceram..

[20] Park S.M., Park J.M., Kim S.K., Heo S.J., Koak J.Y. (2020). Flexural Strength of 3D-Printing Resin Materials for Provisional Fixed Dental Prostheses. Materials.

[21] Keßler A., Dosch M., Reymus M., Folwaczny M. (2022). Influence of 3D-printing method, resin material, and sterilization on the accuracy of virtually designed surgical implant guides. J. Prosthet. Dent..

[22] Alharbi N., Osman R.B., Wismeijer D. (2016). Factors Influencing the Dimensional Accuracy of 3D-Printed Full-Coverage Dental Restorations Using Stereolithography Technology. Int. J. Prosthodont..

[23] Osman R.B., Alharbi N., Wismeijer D. (2017). Build Angle: Does It Influence the Accuracy of 3D-Printed Dental Restorations Using Digital Light-Processing Technology?. Int. J. Prosthodont..

[24] Alharbi N., Osman R., Wismeijer D. (2016). Effects of build direction on the mechanical properties of 3D-printed complete coverage interim dental restorations. J. Prosthet. Dent..

[25] Shim J.S., Kim J.E., Jeong S.H., Choi Y.J., Ryu J.J. (2020). Printing accuracy, mechanical properties, surface characteristics, and microbial adhesion of 3D-printed resins with various printing orientations. J. Prosthet. Dent..

[26] Tahayeri A., Morgan M., Fugolin A.P., Bompolaki D., Athirasala A., Pfeifer C.S., Ferracane J.L., Bertassoni L.E. (2018). 3D printed versus conventionally cured provisional crown and bridge dental materials. Dent. Mater..

[27] Lee E.H., Ahn J.S., Lim Y.J., Kwon H.B., Kim M.J. (2022). Effect of layer thickness and printing orientation on the color stability and stainability of a 3D-printed resin material. J. Prosthet. Dent..

[28] Revilla-León M., Jordan D., Methani M.M., Piedra-Cascón W., Özcan M., Zandinejad A. (2021). Influence of printing angulation on the surface roughness of additive manufactured clear silicone indices: An in vitro study. J. Prosthet. Dent..

[29] Nakai H., Inokoshi M., Nozaki K., Komatsu K., Kamijo S., Liu H., Shimizubata M., Minakuchi S., Van Meerbeek B., Vleugels J. (2021). Additively Manufactured Zirconia for Dental Applications. Materials.

[30] Hofer A.K., Rabitsch J., Jutrzenka-Trzebiatowska D., Hofstetter C., Gavalda-Velasco I., Schlacher J., Schwentenwein M., Bermejo R. (2022). Effect of binder system on the thermophysical properties of 3D-printed zirconia ceramics. Int. J. Appl. Ceram. Technol..

[31] Mei Z., Lu Y., Lou Y., Yu P., Sun M., Tan X., Zhang J., Yue L., Yu H. (2021). Determination of Hardness and Fracture Toughness of Y-TZP Manufactured by Digital Light Processing through the Indentation Technique. BioMed Res. Int..

[32] Revilla-León M., Al-Haj Husain N., Barmak A.B., Pérez-López J., Raigrodski A.J., Özcan M. (2022). Chemical Composition and Flexural Strength Discrepancies Between Milled and Lithography-Based Additively Manufactured Zirconia. J. Prosthodont..

[33] Lu Y., Mei Z., Zhang J., Gao S., Yang X., Dong B., Yue L., Yu H. (2020). Flexural strength and Weibull analysis of Y-TZP fabricated by stereolithographic additive manufacturing and subtractive manufacturing. J. Eur. Ceram. Soc..

[34] Schiltz J., Render T., Gatrell B.A., Qu H., Steiner C., McGinn P., Schmid S. (2020). Wear behavior of additive manufactured zirconia. Procedia Manuf..

[35] Harrer W., Schwentenwein M., Lube T., Danzer R. (2017). Fractography of zirconia-specimens made using additive manufacturing (LCM) technology. J. Eur. Ceram. Soc..

[36] Osman R.B., van der Veen A.J., Huiberts D., Wismeijer D., Alharbi N.M. (2017). 3D-printing zirconia implants; a dream or a reality? An in-vitro study evaluating the dimensional accuracy, surface topography and mechanical properties of printed zirconia implant and discs. J. Mech. Behav. Biomed. Mater..

[37] (2015). Dentistry-Ceramic Materials—Part 4: Types, Classes, and Their Identification.

[38] (1993). Advanced Technical Ceramics—Monolithic Ceramics—General and Textural Properties Part 2: Determination of Density and Porosity.

[39] Noro A., Kaneko M., Murata I., Yoshinari M. (2013). Influence of surface topography and surface physicochemistry on wettability of zirconia (tetragonal zirconia polycrystal). J. Biomed. Mater. Res. B Appl. Biomater..

[40] Opalinska A., Malka I., Dzwolak W., Chudoba T., Presz A., Lojkowski W. (2015). Size-dependent density of zirconia nanoparticles. Beilstein J. Nanotechnol..

[41] Stawarczyk B., Özcan M., Hallmann L., Ender A., Mehl A., Hammerlet C.H. (2013). The effect of zirconia sintering temperature on flexural strength, grain size, and contrast ratio. Clin. Oral. Investig..

[42] Li H., Song L., Sun J., Ma J., Shen Z. (2020). Stereolithography-fabricated zirconia dental prostheses: Concerns based on clinical requirements. Adv. Appl. Ceram..

[43] Roulet J.F., Schepker K.L., Truco A., Schwarz H.C., Rocha M.G. (2021). Biaxial flexural strength, crystalline structure, and grain size of new commercially available zirconia-based ceramics for dental appliances produced using a new slip-casting method. J. Mech. Behav. Biomed. Mater..

[44] Žmak I., Ćorić D., Mandić V., Ćurković L. (2019). Hardness and Indentation Fracture Toughness of Slip Cast Alumina and Alumina-Zirconia Ceramics. Materials.

[45] Bollen C., Lambrechts P., Quirynen M. (1997). Comparison of surface roughness of oral hard materials to the threshold surface roughness for bacterial plaque retention: A review of the literature. Dent. Mater..

[46] Anusavice K.J., Chen C., Rawls H.R. (2013). Phillips Science of Dental Materials.

[47] Revilla-León M., Al-Haj Husain N., Ceballos L., Özcan M. (2021). Flexural strength and Weibull characteristics of stereolithography additive manufactured versus milled zirconia. J. Prosthet. Dent..

[48] Bergler M., Korostoff J., Torrecillas-Martinez L., Mante F.K. (2021). Ceramic Printing—Comparative Study of the Flexural Strength of 3D Printed and Milled Zirconia. Int. J. Prosthodont..

[49] Lerner H., Nagy K., Pranno N., Zarone F., Admakin O., Mangano F. (2021). Trueness and precision of 3D-printed versus milled monolithic zirconia crowns: An in vitro study. J. Dent..

[50] Wang W., Sun J. (2021). Dimensional accuracy and clinical adaptation of ceramic crowns fabricated with the stereolithography technique. J. Prosthet. Dent..

